# Central metabolites and peripheral parameters associated neuroinflammation in fibromyalgia patients: A preliminary study

**DOI:** 10.1097/MD.0000000000033305

**Published:** 2023-03-31

**Authors:** Ye-Ha Jung, Hyeonjin Kim, Seongho Seo, Dasom Lee, Jae-Yeon Lee, Jee Youn Moon, Gi Jeong Cheon, Soo-Hee Choi, Do-Hyung Kang

**Affiliations:** a Department of Psychiatry, Seoul National University Hospital, Seoul, Republic of Korea; b Department of Radiology, Seoul National University Hospital, Seoul, Republic of Korea; c Department of Electronic Engineering, Pai Chai University, Daejeon, Republic of Korea; d Department of Anesthesiology and Pain Medicine, Seoul National University Hospital, Seoul, Republic of Korea; e Department of Nuclear Medicine, Seoul National University College of Medicine, Seoul, Republic of Korea; f Department of Psychiatry, Seoul National University College of Medicine and Institute of Human Behavioral Medicine, SNU-MRC, Seoul, Republic of Korea; g Seoul Chung Psychiatry Clinic, Seoul, Republic of Korea.

**Keywords:** [11C]-(R)-PK11195 PET, creatine, creatinine, fibromyalgia, magnetic resonance spectroscopy (MRS), neuroinflammation

## Abstract

To identify central metabolites and peripheral measures associated with neuroinflammation in fibromyalgia (FM), we scanned [^11^C]-(R)-PK11195 positron emission tomography and magnetic resonance spectroscopy in FM patients. We measured associations between neurometabolite levels measured by magnetic resonance spectroscopy and the extent of neuroinflammation inferred by the distribution volume ratios of [^11^C]-(R)-PK11195 positron emission tomography in 12 FM patients and 13 healthy controls. We also examined the associations between peripheral parameters, such as creatinine and C-reactive protein, and neuroinflammation. In FM patients, we found negative correlations between neuroinflammation and the creatine (Cr)/total creatine (tCr; Cr + phosphocreatine) ratios in the right (r = −0.708, *P* = .015) and left thalamus (r = −0.718, *P* = .008). In FM patients, negative correlations were apparent between neuroinflammation and the glutamate/tCr ratio in the right insula (r = −0.746, *P* = .005). In FM patients, we found negative correlations between neuroinflammation in the left thalamus (r = –0.601, *P* = .039) and left insula (r = −0.598, *P* = .040) and the blood creatinine levels. Additionally, we found significant correlations of other peripheral measures with neuroinflammation in FM patients. Our results suggest that both central metabolites, such as Cr and glutamate, and peripheral creatinine and other parameters are associated with neuroinflammation in patients with FM.

## 1. Introduction

Fibromyalgia (FM) refers to chronic, extensive musculoskeletal pain that may reflect changes in central nervous system (CNS) nociceptive processing.^[1]^ Concomitant symptoms of FM include fatigue, memory problems, and sleep and mood disturbances.^[2]^ Psychological factors such as stress can enhance pain.^[1]^ The clinical features of FM are linked to various psychological symptoms including stress and post-traumatic stress disorder (PTSD).^[3,4]^ Peripheral and central nociceptive abnormalities have been described in FM patients.^[5]^ The heightened pain sensitivity is attributable to peripheral, central, cognitive-emotional, and interpersonal sensitization.^[6]^ Functional and structural brain imaging moderately supported region-specific changes in gray matter volumes, decreased functional connectivity of the descending pain-modulating system, and an increased activity in the pain matrix involved in central sensitization.^[7]^

Central sensitization and neuroinflammation play major roles in FM pathophysiology.^[8]^ Aberrant glial activation takes part in establishment and/or maintenance of central sensitization.^[9]^ Chronic pain is maintained, in part, by central sensitization, which is driven by neuroinflammation of the peripheral and CNSs.^[10]^ A characteristic feature of neuroinflammation is glial cell (microglia and astrocyte) activation in the spinal cord and brain, triggering release of proinflammatory cytokines and chemokines.^[10]^ Glial cell activation plays an important role in the pathogeneses of various neurodegenerative disorders, and also chronic neuropathic pain.^[11]^ Neuroinflammation, characterized by activation of glial cells and the production of inflammatory mediators in the peripheral nervous system and the CNS, plays important roles in the induction and maintenance of chronic pain, suggesting that targeting of excessive neuroinflammation could yield new therapeutic modalities for chronic pain.^[12]^ Neuroinflammation was apparent in the cerebrospinal fluid, and chronic systemic inflammation was evident in the plasma of FM patients.^[13]^ Neuroinflammation revealed by positron emission tomography (PET) reflected brain glial activation, modulation of which might usefully treat FM.^[14,15]^ Therefore, the identification of central metabolites that affect neuroinflammation may aid the development of effective FM treatments. The other potential mechanisms in play include a peripheral nervous system contribution to pain, and systemic inflammation.^[1]^ Thus, identification of FM blood biomarkers would improve FM diagnosis and the development of effective medical treatments.

[^11^C]-(R)-PK11195 is a PET ligand of the translocator protein expressed by activated microglia and astrocytes.^[16]^ Neuroinflammation may be in play if activated microglia or astrocytes are present; these cells exhibit high-level expression of translocator protein.^[16]^ We used [^11^C]-(R)-PK11195 and PET to explore neuroinflammation in FM patients.

Localized proton magnetic resonance spectroscopy (^1^H-MRS) is a noninvasive tool that yields in vivo neurochemical information, principally the levels of human brain metabolites. We used magnetic resonance spectroscopy (MRS) to identify the central metabolites of FM patients. We focused on the levels of creatine (Cr), phosphocreatine (PCr), N-acetylaspartate (NAA), N-acetylaspartylglutamate, glutamine (Gln), glutamate (Glu), myo-inositol, glycerophosphocholine, and glutathione, relative to those of total Cr (tCr) (thus Cr and PCr) in the bilateral thalamus and insula.

We earlier used PET to identify abnormal neuroinflammation^[15]^ and MRS to define abnormal neurometabolites^[17]^ in FM patients. Here, we sought central metabolites and peripheral parameters that might affect such neuroinflammation; we used [^11^C]-(R)-PK11195 PET and MRS to this end.

Various peripheral parameters are known to be associated with inflammation, such as low serum creatinine, increased C-reactive protein (CRP), reduced total and LDL-cholesterol, decreased iron saturation accompanied by high ferritin, erythropoietin resistance, hypoalbuminemia, and frailty.^[18]^ The relationship of serum creatinine with inflammation was also reported in essential hypertension.^[19]^ We previously reported a tendency of low creatinine levels in FM patients.^[20]^ Thus, we also investigated the relationship between creatinine levels and neuroinflammation, and hypothesized that low creatinine levels may be associated with neuroinflammation in FM patients in this study. We explored various other peripheral measures including CRP using routine blood and urine analysis in FM patients.

## 2. Methods

### 2.1. Participants

We used the same participants as the previous report^[17]^ to investigate additional study. We included 12 patients who met the 2010 American College of Rheumatology criteria for FM,^[21]^ recruited in Seoul National University Hospital, and we used online advertisements to recruit 15 controls of comparable age and gender who reported no pain and exhibited no neurological symptoms. However, MRS data were missing for 2 patients; thus, 13 subjects served as healthy controls. Subjects who exhibited high levels of high-sensitivity CRP (hs-CRP) or leukocytosis were excluded. The FM inclusion criteria were as follows: a diagnosis of FM; age between 21 and 63 years; and patients who were not taking benzodiazepine or those who could stop the benzodiazepine medication 2 weeks before the study. Individuals with any major neuropsychiatric disorder prior to FM diagnosis, a neurological disease (cerebrovascular disease or a brain tumor), a history of brain trauma, high levels of hs-CRP or leukocytosis, and who could not undergo magnetic resonance imaging, were excluded. The study was approved by the Institutional Review Board of the Seoul National University Hospital (IRB No. 1703-138-841). Data were obtained only when subjects gave written informed consent after a full explanation of the experimental methods.

As standard clinical tests and observations associated with inflammation include low serum creatinine and increased CRP,^[18]^ we focused on the correlations between creatinine and hs-CRP levels and neuroinflammation in FM patients. We also measured various peripheral parameters which may be associated with neuroinflammation as an exploratory purpose. Routine blood and urine analysis yielded the peripheral data. We obtained white blood cell, segmented neutrophils, absolute neutrophil count, lymphocyte, monocyte, eosinophil, basophil, red blood cell, hematocrit, hemoglobin, mean corpuscular volume, mean corpuscular hemoglobin, mean corpuscular hemoglobin concentration, red blood cell distribution width, platelet, plateletcrit, mean platelet volume, and platelet distribution width using EDTA-treated whole blood. Calcium, phosphorus, glucose, uric acid, cholesterol, serum total protein, albumin, alkaline phosphatase, creatinine, blood urea nitrogen (BUN), total bilirubin, aspartate aminotransferase, alanine aminotransferase, glomerular filtration rate, hs-CRP levels, sodium (Na), potassium (K), chloride (CL), and total CO_2_, were measured from blood serum. The urine pH and specific gravity (SG) were measured.

### 2.2. ^1^H-MRS data acquisition and processing

Four volumes of interest (VOIs) were selected in the right and left thalamus (2 × 2 × 1.5 cm^3^) and right and left insula (2 × 1.5 × 2 cm^3^) for each subject. We focused on the levels of Cr, PCr, NAA, N-acetylaspartylglutamate, Gln, Glu, myo-inositol, glycerophosphocholine, and glutathione, relative to those of tCr in the bilateral thalamus and insula. Following auto-shimming over each VOI, ^1^H-MRS data were acquired using a point-resolved spectroscopy pulse sequence.^[22]^ ^1^H-MRS data were analyzed using LC Model^[23]^ software (ver. 6.3–1J) over the range of 4.2 to 1.0 ppm. Each metabolite level was normalized to that of tCr. The final data analysis included only those metabolites with Cramer–Rao lower bounds < 30%. The number of samples analyzed in terms of each metabolite thus differed among the subjects. The details about MRS data acquisition and processing were presented in our previous report.^[17]^

### 2.3. PET/magnetic resonance image (MRI) acquisition

The same regions of interest of bilateral thalamus and bilateral insula as MRS VOIs were selected for PET/MRI acquisition. Each participant underwent a 60 minutes dynamic [^11^C]-(R)-PK11195 PET scan using a PET/MRI scanner (Biograph mMR, Siemens Healthcare GmbH, Erlangen, Germany). PET was performed using a spatial resolution of 4.4 mm at 1 cm and 5.2 mm full-width at half-maximum, with a 10 cm offset from the center of the transverse field of view. The details were described in the authors previous study.^[24]^

### 2.4. Binding quantification

A parametric image of the distribution volume ratio (DVR) for each subject was generated via relative equilibrium-based graphical analysis using a reference region.^[25]^ The regions of interest DVR values were then extracted from the normalized DVR images with the aid of population-based probability maps.^[26,27]^ The details were described in the authors previous study.^[15,24]^

### 2.5. Synthesis of [^11^C]-(R)-PK11195

[^11^C]-(R)-PK11195 was synthesized using a previously reported method with few modifications.^[28]^ The details were described in the authors previous study.^[15]^

### 2.6. Statistical analysis

All statistical analyses were performed using SPSS ver. 21.0 software (IBM Corporation, Armonk, NY). Pearson correlation analysis was used to explore the associations between pairs of variables. A *P* value < .05 was considered to reflect significance.

## 3. Results

### 3.1. Study participants and basic information

A total of 12 FM patients and 13 healthy controls completed the study. Their demographic and clinical characteristics are listed in Table 1. None of age, gender ratio, or educational level differed significantly between the 2 groups. The average pain duration in FM patients was 4.4 years.

**Table 1:** Participant’s demographic characteristics.

	FM patients	Healthy controls	*P* value
N	12	13	
Age	41.7 ± 14.0	40.1 ± 6.3	*P* = .723
Gender	5M, 7F	9M, 4F	*P* = .165
Education (yr)	14.4 ± 2.6	16.2 ± 2.1	*P* = .068
Duration of illness (yr)	4.4 ± 3.1	N/A	

Data are mean ± standard deviation.

F = female, FM = fibromyalgia, M = male.

### 3.2. Correlations between neuroinflammation inferred by the DVRs of [^11^C]-(R)-PK11195 and central metabolite levels

**Figure 1.**
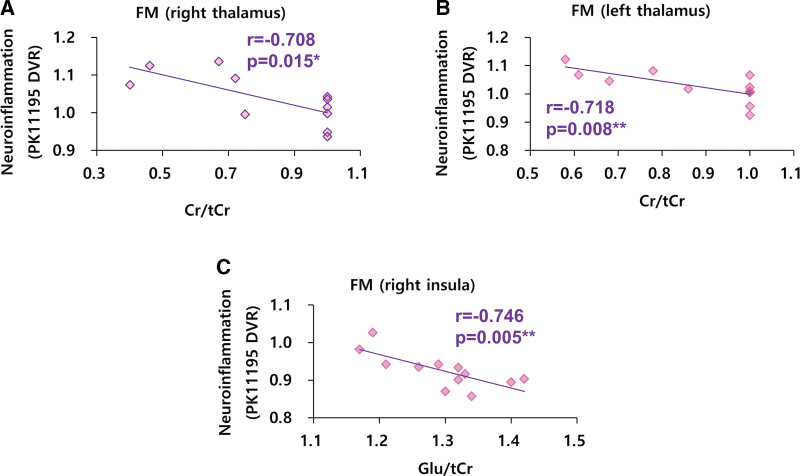
Correlations between central metabolites and neuroinflammation (PK11195 DVR) in thalamus and insula of FM patients. (A–C), (A) n = 11, (B and C) n = 12. DVR = distribution volume ratio, FM = fibromyalgia, Cr = creatine, tCr = total creatine, Glu = glutamate.

In patients with FM, we found negative correlations between neuroinflammation and the Cr/tCr ratio in the right thalamus (r = −0.708, *P* = .015, Fig. 1A) and the left thalamus (r = −0.718, *P* = .008, Fig. 1B). In FM patients, negative correlations were evident between neuroinflammation and the Glu/tCr ratio in the right insula (r = −0.746, *P* = .005, Fig. 1C). Except these findings, other significant correlations between neuroinflammation and central metabolites were not found in FM patients.

We found positive correlations between neuroinflammation and the Glu/tCr ratio in the right thalamus of healthy controls (*R* = 0.661, *P* = .019). We found negative correlations between neuroinflammation and the Gln/tCr ratio in the right insula of healthy controls (r = −0.954, *P* = .003). We found positive correlations between neuroinflammation and the NAA/tCr ratio in the right insula of healthy controls (*R* = 0.678, *P* = .022). Except these results, other significant correlations between neuroinflammation and central metabolites were not found in healthy controls.

### 3.3. Correlations between neuroinflammation inferred by the DVRs of [^11^C]-(R)-PK11195 and peripheral measures

**Figure 2.**
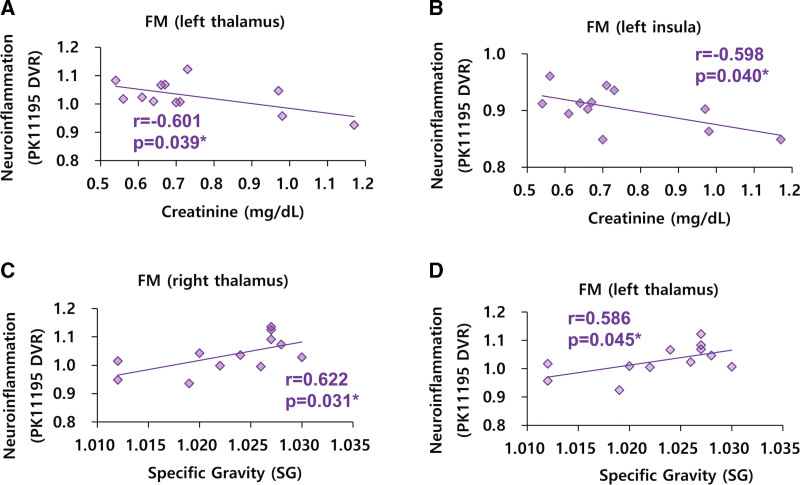
Correlations between peripheral parameters and neuroinflammation (PK11195 DVR) in FM patients. (A–D) n = 12. DVR = distribution volume ratio, FM = fibromyalgia.

**Figure 3.**
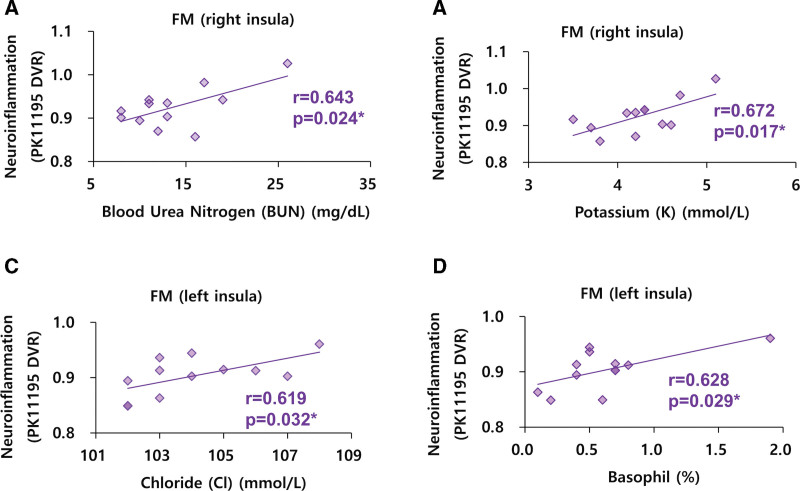
Correlations between peripheral parameters and neuroinflammation (PK11195 DVR) in FM patients. (A–D) n = 12. DVR = distribution volume ratio, FM = fibromyalgia.

In patients with FM, we found negative correlations between neuroinflammation in the left thalamus and the blood creatinine level (r = −0.601, *P* = .039, Fig. 2A). We found negative correlations between neuroinflammation in the left insula and the blood creatinine level (r = −0.598, *P* = .040, Fig. 2B) in FM patients. We found positive correlations between neuroinflammation in the right thalamus and the SG (*R* = 0.622, *P* = .031, Fig. 2C) in FM patients. We found positive correlations between neuroinflammation in the left thalamus and the SG (*R* = 0.586, *P* = .045, Fig. 2D) in FM patients. We found positive correlations between neuroinflammation in the right insula and the BUN (*R* = 0.643, *P* = .024, Fig. 3A) in FM patients. We found positive correlations between neuroinflammation in the right insula and the serum potassium level of FM patients (*R* = 0.672, *P* = .017, Fig. 3B). We found positive correlations between neuroinflammation in the left insula and the serum chloride level (*R* = 0.619, *P* = .032, Fig. 3C) in FM patients. We found positive correlations between neuroinflammation in the left insula and the blood basophil level (*R* = 0.628, *P* = .029, Fig. 3D) in FM patients. Except these findings, other significant correlations between neuroinflammation and peripheral parameters were not found in FM patients.

We found negative correlations between neuroinflammation in the right thalamus and the blood monocyte level (r = −0.621, *P* = .024) in healthy controls. We found negative correlations between neuroinflammation in the right insula and the mean corpuscular hemoglobin concentration level (r = −0.555, *P* = .049) in healthy controls. Except these results, other significant correlations between neuroinflammation and peripheral parameters were not found in healthy controls.

## 4. Discussion

In short, central metabolites associated with neuroinflammation were Cr/tCr and Glu/tCr in the right and left thalamus and the right insula in FM patients. Peripheral measures associated with neuroinflammation in thalamus and insula were creatinine, BUN, potassium, chloride and basophil in blood and urine SG in FM patients. [^11^C]-(R)-PK11195 PET revealed abnormal neuroinflammatory levels in the brains of FM patients; such neuroinflammation may contribute to FM pathology.^[15]^ First of all, central metabolite affecting neuroinflammation was Cr/tCr in the right and left thalamus in FM patients. In such patients, low Cr/tCr ratio correlated with high-level neuroinflammation in the right and left thalamus. Creatine is a key regulator of brain energy homeostasis; balanced Cr metabolism is essential to ensure appropriate brain functioning and neurotransmission.^[29,30]^ As Cr which is highly concentrated in muscle and brain tissues plays its main roles in energy metabolism and cytoprotection,^[30]^ low Cr levels in brain may be associated with dysfunctional energy metabolism^[31]^ and elevated neuroinflammation in FM patients, contributing to FM pathology.

In addition, the high levels of PTSD were related to the low levels of Cr/tCr ratio in the right and left thalamus of FM.^[31]^ Also, the increases of PTSD and stress were associated with the increase of neuroinflammation in FM.^[15]^ Thus, PTSD and stress may be associated with the decrease of Cr, which may contribute to the increase of neuroinflammation in FM. As prolonged stress reduced Cr concentrations in the rat medial prefrontal cortex,^[32]^ and as neuroinflammation elevated microglial activity that in turn triggered stress,^[33]^ psychological conditions may increase neuroinflammation and reduce Cr levels in FM patients. Environmental enrichment after mild traumatic brain injury decreased neuroinflammation, and increased brain energy homeostasis and cognitive functioning.^[34]^ Improvement in brain Cr levels and energy homeostasis may reduce FM-associated neuroinflammation. Brain Cr levels reflect both dietary Cr concentrations and endogenous Cr synthesis.^[29]^ Thus, Cr supplementation may balance brain Cr metabolism and reduce neuroinflammation in FM patients. Creatine supplementation is a safe, effective, and tolerable adjunct to medications used to treat neurological disorders associated with dysfunctional energy metabolism.^[35]^ Furthermore, Cr supplementation has been reported to be a useful dietary intervention to improve muscle function in FM patients.^[36]^ Thus, Cr supplementation may contribute to the decrease of neuroinflammation in FM patients. In particular, considering that Cr synthesis primarily occurs in the liver and kidneys,^[37]^ the decrease of Cr may be associated with renal function, which may contribute to the increase of neuroinflammation in the right and left thalamus of FM.

Low Glu/tCr ratio was associated with high-level neuroinflammation in the right insula of FM patients. As prolonged stress decreased both Glu and Cr concentrations in the rat medial prefrontal cortex,^[32]^ such decreases may increase neuroinflammation in FM patients. On the other hand, we found positive correlations between neuroinflammation and the Glu/tCr ratio in the right thalamus of healthy controls. Glutamate is the major excitatory neurotransmitter of the CNS; extrasynaptic glutamate diffusion is strongly associated with glial reaction and neuroinflammation.^[38]^ Thus, high Glu levels may increase neuroinflammation in healthy controls but reduce neuroinflammation in FM patients. These contrary results between healthy controls and FM patients require further study.

We found significant correlations between neuroinflammation and the blood creatinine, BUN, potassium, chloride, and basophil levels and urine SG in FM patients. Low creatinine levels may be the prime peripheral biomarker of neuroinflammation in FM patients. Low creatinine levels were also associated with increased neuroinflammation in patients with complex regional pain syndrome.^[39]^ FM patients exhibited significantly lower urinary creatinine excretion than did patients with major depression.^[40]^ The creatinine level tended to be lower in FM patients than healthy controls, and a low creatinine level correlated with high-level affective pain in FM patients.^[20]^ Also, the serum creatinine levels were significantly reduced in FM patients compared to the healthy controls (*P* = .029) in this study. Thus, a low creatinine level may be a key biomarker of FM pathology and high-level neuroinflammation. Creatinine production requires Cr, PCr, and adenosine triphosphate; creatinine is a muscle-stored degradation product of Cr.^[41]^ Considering that Cr is spontaneously converted to creatinine, the decrease of Cr may be related to the reduction of creatinine in FM patients. Serum creatinine is widely used clinically as an index of renal function.^[42,43]^ In addition, low serum creatinine is known to be associated with inflammation.^[18]^ Thus, the decrease of serum creatinine may be associated with inflammation related to renal dysfunction,^[44]^ which may contribute to the increase of neuroinflammation in the left thalamus and insula of FM patients.

The increase of BUN significantly correlated with the increased neuroinflammation in the right insula of FM patients. BUN levels were increased in a chronic kidney disease animal model.^[45]^ Thus, the increase of BUN may be associated with renal function and neuroinflammation in FM patients. The decrease of Cr/tCr ratio in the right thalamus significantly correlated with the increase of SG in FM patients (r = −0.734, *P* = .010). SG was significantly higher in FM patients compared to healthy controls in this study (*P* = .016). The increase of SG was associated with the increase of neuroinflammation in right and left thalamus in FM patients. Urine SG could be used to detect kidney impairment.^[46]^ Thus, the increase of SG may be related to renal function, which may contribute to the increase of neuroinflammation in the right thalamus in FM patients. Stress and renal function^[47]^ may contribute to the increase of potassium, which may be associated with the increase of neuroinflammation in the right insula in FM patients. Changes in serum chloride concentration are associated with increased risk of acute kidney injury.^[48]^ The increase of basophil significantly correlated the increased neuroinflammation in the left insula in FM patients. The bidirectional interaction between these 3 immune cell types (mast cells, eosinophils and basophils) and the nervous system is involved in the pathogenesis of neurogenic inflammation, pain and pruritus.^[49]^ Stress-related physiological mechanisms are seen as upstream drivers of neurogenic inflammation in FM.^[50]^ In addition, altered basophil function was found in patients with chronic kidney disease.^[51]^ Both corticotrophin releasing factor and adrenocorticotrophic hormone were shown to activate basophils,^[52]^ showing hypothalamic-pituitary-adrenal axis-related stress effects on basophil function. Basophils produce IL-6,^[53]^ and IL-6 is associated with neuroinflammatory cascade across schwann cell-neuron-microglia.^[54]^ Thus, the increase of basophils may contribute to the increased neuroinflammation in FM patients.

Low serum creatinine is known to be associated with inflammation,^[18]^ and the present study is first report showing the possible association of low serum creatinine with neuroinflammation in FM patients. Although they were exploratory findings, we also found significant associations of blood BUN, potassium, chloride, and basophil levels and urine SG with neuroinflammation in FM patients. Our work had certain limitations, principally the small number of FM patients. Future studies with larger sample sizes are required. Although we found significant correlations between neuroinflammation and central metabolites and peripheral measures in FM patients, it was not easy to infer and discuss hidden mechanisms possible to explain the relationship between them, based on only the correlations. As this is an exploratory and preliminary study, future studies able to confirm the relationship between them are required.

## 5. Conclusions

In conclusion, we suggest that characterizing the central and peripheral biomarkers that affect neuroinflammation is essential to understanding the pathology of FM patients. First of all, the decrease of Cr was related to the increase of PTSD in FM,^[31]^ which may contribute to the increase of neuroinflammation in FM patients. And, the end product of Cr metabolism, creatinine was reduced in FM patients, which may contribute to the increase of neuroinflammation in FM patients. Central Cr and Glu and peripheral creatinine, BUN, K, CL, basophil and SG all may be associated with renal function. Patients with chronic kidney disease present with systemic chronic inflammation, which promotes neuroinflammation in brain tissue.^[45]^ Stress can have implications for kidney disease, its progression, and its complications through multiple stressors and pathways.^[47]^ Psychological stress is associated with FM,^[55]^ and the increases of PTSD and stress were associated with the increase of neuroinflammation in FM patients.^[15]^ Thus, stress-mediated renal dysfunctions may be associated with abnormal levels of Cr, Glu, creatinine, BUN, K, CL, basophil and SG, which may contribute to the increase of neuroinflammation in FM patients.

## Acknowledgments

The authors wish to thank all participants for their valuable time engaging with this research.

## Author contributions

**Conceptualization:** Ye-Ha Jung.

**Data curation:** Seongho Seo, Dasom Lee, Jae-Yeon Lee.

**Formal analysis:** Ye-Ha Jung.

**Funding acquisition:** Soo-Hee Choi, Do-Hyung Kang.

**Investigation:** Ye-Ha Jung, Hyeonjin Kim, Seongho Seo.

**Methodology:** Hyeonjin Kim, Seongho Seo.

**Project administration:** Dasom Lee, Do-Hyung Kang.

**Supervision:** Soo-Hee Choi, Do-Hyung Kang.

**Visualization:** Dasom Lee.

**Writing – original draft:** Ye-Ha Jung.

**Writing – review & editing:** Ye-Ha Jung, Jee Youn Moon, Gi Jeong Cheon, Soo-Hee Choi, Do-Hyung Kang.
